# FNAC diagnosis of medullary carcinoma thyroid: A report of three cases with review of literature

**DOI:** 10.4103/0970-9371.70745

**Published:** 2010-04

**Authors:** Ghazala Mehdi, Veena Maheshwari, Hena A Ansari, Lubna Sadaf, Mohammad Amanullah Khan

**Affiliations:** Department of Pathology, Jawaharlal Nehru Medical College, Aligarh Muslim University, Aligarh, Uttar Pradesh, India; 1 Department of Surgery, Jawaharlal Nehru Medical College, Aligarh Muslim University, Aligarh, Uttar Pradesh, India

**Keywords:** Medullary thyroid carcinoma, fine needle aspiration cytology, plasmacytoid type, spindle cell type

## Abstract

Medullary carcinoma of the thyroid is an unusual neoplasm, which is associated with specific supportive diagnostic markers. Despite this, its cytological diagnosis is often difficult. We report herewith three cases of medullary thyroid carcinoma. The diagnosis was established on fine-needle aspiration cytology. Plasmacytoid cell pattern was observed in two cases and spindle cell pattern in the third case.

## Introduction

Medullary thyroid carcinoma is a tumor of the parafollicular C-cells. This tumor merits special attention because detection of the precursor lesion (C-cell hyperplasia)[1] and the hallmark genetic mutation in the RET gene[2] in specific cases can actually enable the prevention of this tumor. Monitoring of patients with a positive family history is, therefore, of great importance. We present three cases of medullary thyroid carcinoma, which were diagnosed on cytology, along with a short review of literature.

## Case Reports

### Case 1

A 70-year-old female patient presented with a progressively increasing midline neck swelling for the past 10 years, hoarseness of voice, dysphagia and generalized weakness for 5 months. Physical examination revealed an oval, firm to hard swelling in the midline of neck, moving with deglutition and an enlarged left-sided cervical lymph node.

### Case 2

The second patient was a 55-year-old male with a large swelling extending from the midline to the left side of the neck since 2 years. The patient complained of dyspnoea, dysphagia and hoarseness of voice for the last 20 days. Examination showed a firm to hard neck mass, occupying the entire anterior and posterior triangles of the neck and moving with deglutition. Two small cervical lymph nodes were detected on the left side, posterior, to the sternocleidomastoid muscle.

### Case 3

A 40-year-old male presented with a left-sided neck mass since 1 year, associated with hoarseness of voice for the past 15 days. On examination, 2 lumps were noted, one in the midline measuring 3×3 cm and the other on the left side, measuring 1×1 cm. Both were firm to hard and moved with deglutition. No cervical lymph node was palpable.

There was no family history of similar tumors in any of the 3 patients and none had any other endocrine disorder.

Fine-needle aspiration cytology (FNAC) from the neck masses and enlarged lymph nodes were obtained. The smears were stained by Papanicolaou (Pap) and Hematoxylin and Eosin (H and E) stains.

**Figure 1:**
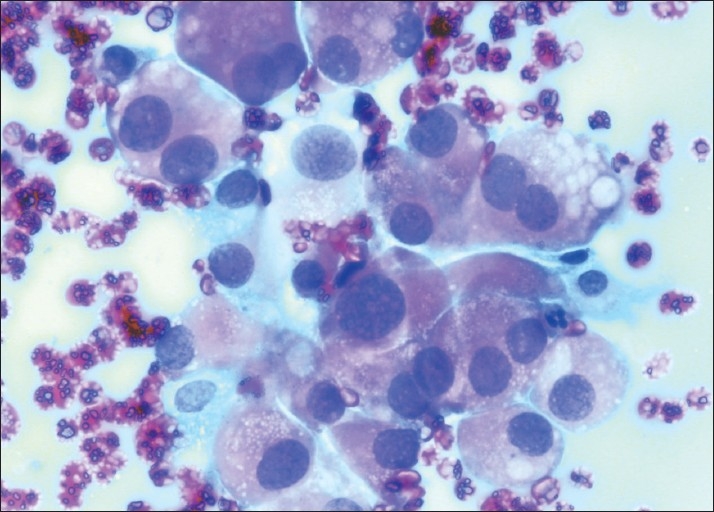
FNAC smears show plasmacytoid cells with abundant cytoplasm and eccentric pleomorphic nuclei (Pap, ×400)

**Figure 2:**
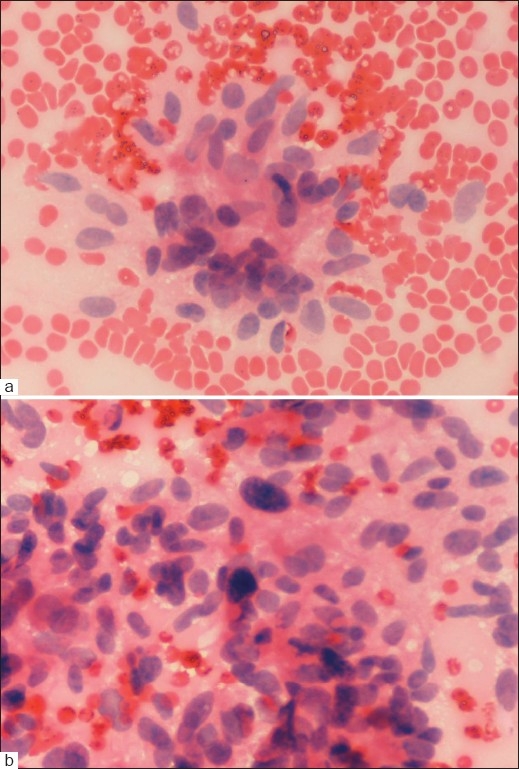
(a, b) FNAC smears show cluster of spindle tumour cells with intervening amorphous eosinophilic material (H and E, ×400)

Microscopic examination of the smears in the first 2 cases showed clusters as well as singly dispersed plasmacytoid cells of variable sizes. The cells had eccentric nuclei with coarse chromatin and abundant amphophilic cytoplasm [Figure 1]. Small clumps of amyloid-like amorphous, glassy, eosinophilic material were seen in the background. A diagnosis of the medullary thyroid carcinoma (plasmacytoid type) was made on the basis of these cytological findings. Aspirates from the third case showed several small and large clusters of elongated spindle-shaped cells with scant cytoplasm and hyperchromatic, pleomorphic nuclei. Scant, glassy pink amyloid-like material was present in this case as well [Figure 2a, b]. Based on these cytological features, a diagnosis suggestive of medullary thyroid carcinoma (spindle cell variant) was rendered.

Smears from cervical lymph nodes in the first 2 cases showed infiltration of lymphoid tissue with cells similar to those seen in the respective tumor aspirates, establishing the presence of metastasis.

## Discussion

Medullary thyroid carcinoma is the first human malignancy known to be associated with a tumor marker, the hormone calcitonin, measurement of which enables diagnosis as well as prognostication, following surgical resection of the primary thyroid tumor.[3] In familial cases, identification of the precursor lesion of this tumor and genetic mutation allows for early diagnosis and therapy. It is also associated with other endocrine abnormalities, including parathyroid hyperplasia and pheochromocytoma, as part of the multiple endocrine neoplasia (MEN) syndromes.[4]

Children are almost exclusively afflicted by the familial variant.[1] The gene involved in the development of this tumor is the RET gene, located on chromosome 10q11.2, which is affected by activating germline mutations.[2] RET mutations have also been detected in sporadic medullary thyroid carcinoma, but in contrast to the familial and MEN-related cases, these mutations are somatic, that is, found only in the tumor cells.[2]

FNAC smears from the plasmacytoid medullary thyroid carcinoma are usually cellular, yielding tumor cells that are dispersed and are characterized by eccentric nuclei, “neuroendocrine type” chromatin, inconspicuous nucleoli, binucleated and multinucleated cells and a relatively clean background.[5] The cytoplasm of the tumor cells is faintly granular in fixed material, but may show conspicuous red granules in air-dried MGG-stained smears.[6]

Apart from the classic plasmacytoid cell pattern, the neoplastic cells may resemble spindle cells or small cells with scanty cytoplasm and moulding of nuclei.[6] In such cases, the detection of amyloid is a valuable pointer to the diagnosis. Congo Red staining helps to differentiate amyloid from colloid or hyaline fragments,[6] and is diagnostic for medullary thyroid carcinoma, except in rare cases of primary amyloidosis involving the thyroid[7] and amyloid-producing thyroid plasmacytoma.[8]

Depending on the specific cytomorphology of the tumor, a number of differential diagnoses may arise. The small cell pattern may be mistaken for a malignant lymphoma, poorly differentiated insular carcinoma or metastatic small cell carcinoma, whereas the spindle cell tumor may mimic a fibroblastic tumor or even a melanoma.[6] In such problematic cases, measurement of serum calcitonin levels are very helpful in resolving the dilemma.

An interesting new technique has been described, which is based on the measurement of calcitonin levels in FNAC washout fluid.[9] Another experimental technique has recently been reported in which the tumor cells have been made to fluoresce in vivo.[10] It is proposed that this may help in adequate resection at surgery.[10]

FNAC, although not a substitute for conventional surgical histopathology, is considered a firstline diagnostic test for evaluation of thyroid lesions along with immunocytochemistry. Early diagnosis and treatment would lead to a markedly improved cure rate of these neoplasms.
